# The Association of Growth Differentiation Factor-15 with Left Ventricular Hypertrophy in Hypertensive Patients

**DOI:** 10.1371/journal.pone.0046534

**Published:** 2012-10-11

**Authors:** Hao Xue, Zhenhong Fu, Yundai Chen, Youhong Xing, Jie Liu, Hang Zhu, Xiao Zhou

**Affiliations:** Department of Cardiology, Chinese People's Liberation Army General Hospital, Beijing, People's Republic of China; Universidade Federal do Rio de Janeiro, Brazil

## Abstract

Growth differentiation factor-15 (GDF-15) has been identified as an endogenous anti-hypertrophy effect. However, the association of plasma GDF-15 levels with left ventricular hypertrophy (LVH) in hypertension is poorly understood. We investigate the effect of plasma GDF-15 levels on left ventricular hypertrophy (LVH) in hypertension. We measured the plasma levels of GDF-15 in 299 untreated hypertensive patients which consisted of 99 with LVH and 200 without LVH using immunoradiometric assay. All subjects were examined by the ultrasonic cardiograph to determine Left ventricular (LV) internal diameters, septal thickness, and posterior wall thickness. The associations of GDF-15 with left ventricular mass index (LVMI), LV end-systolic and –diastolic diameters, LV wall thickness, and LV ejection fraction were evaluated. We found that plasma GDF-15 levels in hypertensive patients with LVH [median 1101, 25th–75th percentiles (879–1344) ng/L] were higher than that in hypertensive patients without LVH [median 516, 25th–75th percentiles (344–640) ng/L] (P<0.001). After adjustment for traditional covariates, plasma GDF-15 levels were independently related to LVMI (R^2^ = 0.53; β = 0.624, P<0.001), LV interventricular septal thickness (R^2^ = 0.23; β = 0.087, P<0.01) and LV posterior wall thickness (R^2^ = 0.26; β = 0.103, P<0.05). Our cross-sectional data on a hospital-based sample indicate that plasma GDF-15 levels are associated with LVH in hypertensive patients.

## Introduction

Hypertensive left ventricular hypertrophy (LVH) is the most common target organ damage, which is preclinical cardiovascular disease. Epidemiological studies show that the prevalence of LVH in hypertensive patients was about 25% to 35% in China [1], [2]. It has been shown that LVH increases the risk of stroke, coronary heart disease, congestive heart failure, arrhythmias and sudden cardiac death, which is associated with cardiovascular morbidity and mortality, as well as all-cause mortality [3], [4]. Although hypertension is a major cause of LVH, it is influenced by other traditional cardiac risk factors such as age, sex, life style and diabetes. In addition, some growth factors and cytokines also play important role in the development of LVH in hypertension [5].

Growth differentiation factor (GDF-15) belongs to the transforming growth factor-cytokine superfamily, which originally identified in activated macrophages [6]. GDF-15 is a stress-responsive cytokine and only appreciably expressed in liver and placenta at baseline [7]–[9]. Although GDF-15 is not normally expressed in heart under physiological conditions, it increases rapidly in response to cardiovascular injury, such as pressure overload, heart failure, ischemia/reperfusion, and atherosclerosis [10], [11]. Furthermore, recent studies have reported that GDF-15 is emerging as an independent prognostic biomarker in patients with cardiovascular disease [12]–[14].

Given the rapid change of GDF-15 expression level in response to pressure overload and the important role of GDF-15 in the regulation of cardiac remodeling, we hypothesized that the circulating level of GDF-15 may involved in the development of left ventricular hypertrophy in hypertension. To test our hypothesis, we investigated the potential relationship of plasma GDF-15 levels with measures of left ventricular remodeling in patients with hypertension.

## Methods

### Subjects

Patient recruitment was consecutive from the hypertensive outpatients of the Chinese People's Liberation Army General Hospital. Originally, a total of 299 untreated hypertensive patients were recruited to this study from May 2008 to June 2009.

Blood pressure was measured by professional doctors with a standardized mercury sphygmomanometer after at least 5 min rest in the sitting position at the subject's right upper arm. Three readings were recorded at least 30 seconds apart. The average was used for analysis. Hypertension was defined according to World Health Organization (WHO) criteria [15]: in brief, hypertension was defined as a mean of 3 independent measures of blood pressure ≥140/90 mmHg or current use of antihypertensive drugs. Exclusion criteria included secondary arterial hypertension, atria-ventricular conduction block, chronic obstructive bronchitis, bronchial asthma, chronic myeloproliferative diseases, diabetes, hypertrophic cardiomyopathy, valvular heart diseases, pulmonary hypertension, coronary heart disease and heart failure. The exclusion criteria of coronary heart disease included one of the following: the patient had a history of angina, previous myocardial infarction or myocardial revascularization procedures; or ischemic, pathological Q-waves on electrocardiography or echocardiographic segmental wall motion abnormalities. The exclusion criteria was a diagnosis of heart failure (class II, III or IV according to New York Heart Association criteria or left ventricular ejection fraction <50% in Echocardiogram). All participants were Han nationality.

### Biochemical variables determination and clinical data collection

Blood samples were collected after overnight fasting and analyzed for serum sodium, potassium, creatinine, uric acid, blood urea nitrogen (BUN), total plasma cholesterol (TC), triglyceride (TG), high density lipoprotein cholesterol (HDL-C), low density lipoprotein cholesterol (LDL-C) and blood glucose with an automatic analyzer (Hitachi 7060, Hitachi, Japan). A complete medical history was obtained from all subjects, including family history of hypertension, diabetes mellitus, coronary heart disease or stroke. The following conventional cardiovascular risk factors were also recorded, including alcohol intake, cigarette smoking, and body mass index (BMI). BMI was calculated by using the formula of weight (kg)/height (m^2^). The estimated glomerular filtration rate (eGFR) was estimated with the re-expressed four-variable Modification of Diet in Renal Disease (MDRD) equation [16]:
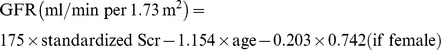



### Echocardiography measurement

Echocardiography was performed in all hypertensive patients (HP 5500, Phillips Medical System, Boston, MA, USA; or an HDI 3000, ATL, Bothell, WA, USA). The transducer frequency was 2.5 to 3.5 MHz. M-dimensional and bi-dimensional echocardiography was recorded at 30 frames per second on super TDK videotape (Tokyo, Japan). Subjects were examined in the supine and left lateral position by three experienced investigators who were blind to the patient's lab tests. Echocardiography images were obtained in the para-sternal long- and short-axis views, and apical two- and four- chamber views with standard transducer positions [17]. Three physician-echocardiographers supervised the echocardiography examination.

Left ventricular internal diameters, septal thickness, and posterior wall thickness were measured on up to 3 cardiac cycles at end-diastole and end-systole according to the recommendations of the American Society of Echocardiography [18]. The LV mass was calculated at end diastole by use of the cube formula:
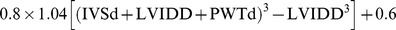
Which yields values closely related (R = 0.90) to necropsy LV weight [19]. IVSd is septal wall thickness, PWTd is posterior wall, and LVIDD is left ventricular end-diastolic diameter. LVM was divided by height 2.7 to obtain LV mass index (LVMI/h^2.7^). LVH was defined as LVMI >49.2 g/m^2.7^ for men and >46.7 g/m^2.7^ for women [20]. Relative wall thickness was calculated by using the formula of 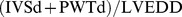, where LVEDD is left ventricular end-diastolic diameter [21]. Echocardiographic variables (IVSd, PWTd, LVEDD, LVEDD) in 10% patients were repeated measures 2 times. The intra-assay and inter-assay variability of the echocardiographic variables including IVSd, PWTd, LVEDD, LVESD are 3.2%, 4.7%, 3.9%, 5.1% and 6.4%,7.9%, 7.1%, 7.7% respectively.

### Determination of plasma GDF-15 levels

The GDF-15 concentrations were determined in hypertensive patients. Blood samples for the determination of GDF-15 were collected on ice in tubes containing EDTA and aprotinin and were centrifuged at 3000 g for 15 min at 4°C to isolate plasma. Plasma samples were then frozen at −70°C. The plasma GDF-15 assay was performed within 3 months using immunoradiometric assay as previously described by an investigator blinded to patient characteristics [22]. The intra-assay and inter-assay variability of the GDF-15 are 5.9% and 8.1% respectively.

### Statistical Analysis

Continuous variables were presented as mean ± standard deviation (SD) or median (with interquartile range), and dichotomous variables as numbers and percentages. GDF-15 was analyzed as continuous variables. Differences in baseline levels of characteristics between hypertensive cases with and without LVH were analyzed with chi-square or *t* tests; the Wilcoxon two-sample test was used as appropriate (for continuous variables that were not normally distributed, such as levels of GDF-15). Receiver operating characteristic (ROC) curves of continuous untransformed GDF-15 were constructed for discrimination between hypertensive patients with or without left ventricular hypertrophy. The areas under the curve (AUC) were compared by using Hanley and McNeil method. P<0.05 were considered significant. We assessed the associations of the plasma GDF-15 levels with measures of LVH by means of linear regression models. Multiple regression models were adjusted for age, sex, BMI, SBP, DBP, TC, LDL-C, HDL-C, TG, eGFR and glucose in hypertensive patients with LVH. In addition, we performed a multivariate logistic regression analysis with the dependent variable as hypertension with and without left ventricular hypertrophy, adjusting by the covariates age, sex BMI, SBP, DBP, TC, LDL-C, HDL-C, TG, eGFR and glucose in hypertensive patients. All statistical analyses were performed using SPSS software version 11.0 (SPSS Inc, Chicago). A 2-sided value of P<0.05 was considered significant.

## Results

### Characteristics of the Subjects

Among the 299 hypertensive patients, a total of 99 subjects were diagnosed as LVH according to the measurement of LVMI. The clinical characteristics of the subjects with and without LVH are shown in Table 1. No difference was found in the mean age, systolic blood pressure, diastolic blood pressure, body mass index, triglyceride, total plasma cholesterol, glucose, eGFR and low density lipoprotein cholesterol in hypertensive patients with or without LVH (Table 1).

**Table 1:** Clinical Characteristics.

Characteristics	Hypertension patients without LVH n = 200	Hypertension patients with LVH n = 99
Age, y	54.9±8.6	57.1±9.1
Men,%	46.8%	50.7%
BMI(kg/m^2^)	23.8±2.90	22.9±3.21
SBP, mmHg	159.0±17.9	163.1±20.9
BP, mmHg	96.6±10.8	99.3±12.1
HDL-C, mmol/L	1.26±0.39	1.33±0.40
LDL-C, mmol/L	3.09±0.58	3.14±0.66
TC, mmol/L	5.12±0.92	5.20±1.01
TG, mmol/L	1.58±1.41	1.49±1.36
eGFR, mL/min/1.73 m^2^	93.2±10.76	91.6±9.78
Glucose.mmol/L	5.18±1.01	5.24±0.98

BMI indicates body mass index. Clinical characteristics of age, BMI, SBP, DBP, eGFR, glucose, HDL-C, LDL-C, TG and TC values are given as mean (SD); categorical variables are presented as percentages.

### GDF-15 and LVH

**Figure 1:**
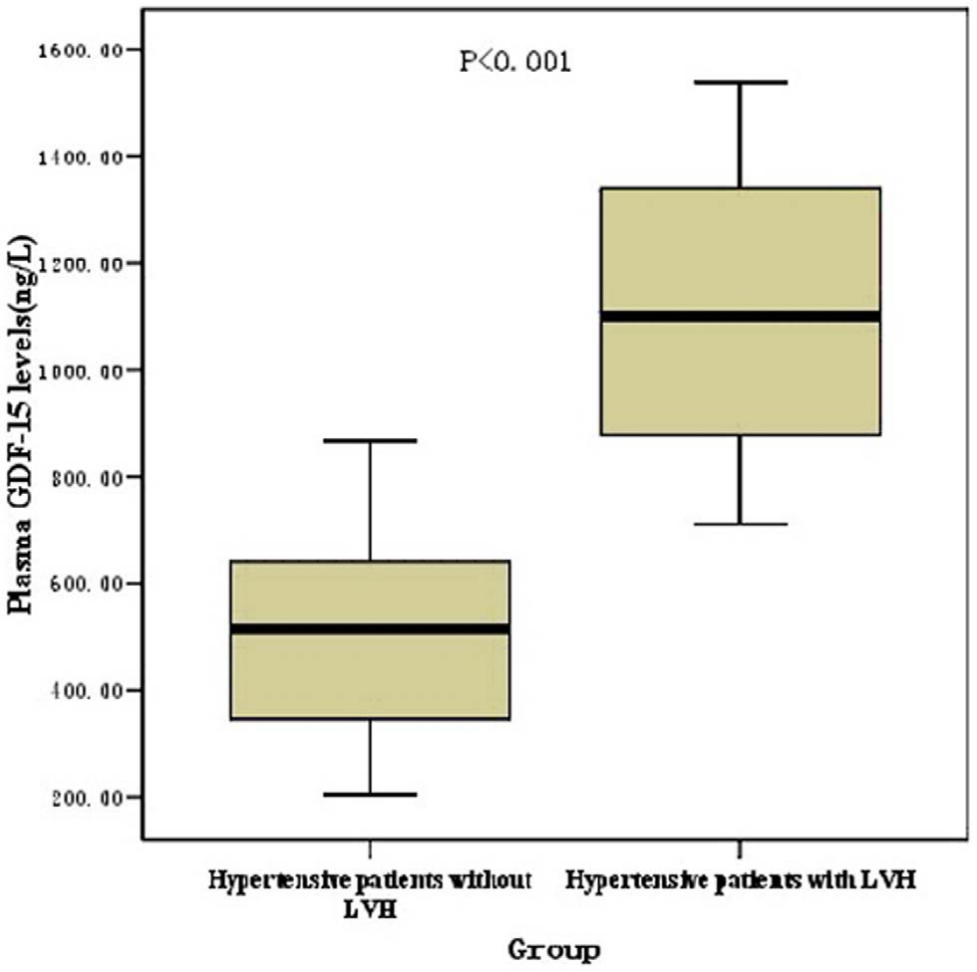
Plasma GDF-15 levels in hypertensive patient with or without LVH. The GDF-15 levels are presented as box (median, 25th percentile, 75th percentile) and whisker (5th and 95th percentiles) plots. P<0.001 by the Wilcoxon two-sample test in panels. GDF-15, growth differentiation factor; LVH, left ventricular hypertrophy.

**Figure 2:**
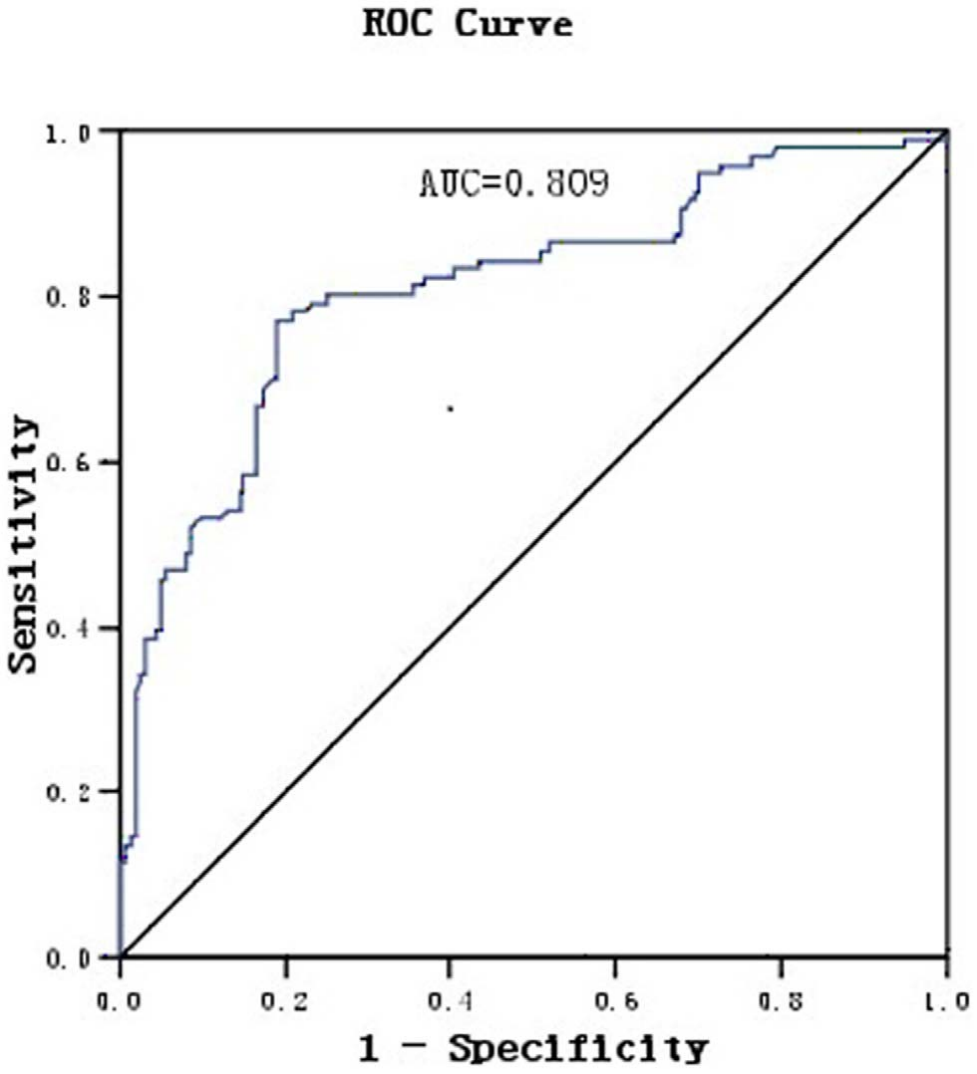
ROC curves for GDF-15 in hypertensive patient with or without LVH.

Plasma GDF-15 levels in hypertensive patients with LVH [median 1101, 25th–75th percentiles (879–1344) ng/L] were higher than that in hypertensive patients without LVH [median 516, 25th–75th percentiles (344–640) ng/L] (Fig. 1). ROC curves for GDF-15 were constructed for discrimination between hypertensive patients with or without left ventricular hypertrophy. The AUC for continuous untransformed GDF-15 is 0.809 [95%CI (confidence interval), (0.753; 0.865)] (p<0.01) (Fig. 2).

After adjusted for age, sex, BMI, SBP, DBP, blood glucose, eGFR, TC, LDL-C, HDL-C and TG in multiple linear regression analysis, plasma GDF-15 levels remained independently associated with increased left ventricular mass index (LVMI) (R^2^ = 0.53; β = 0.624, P<0.001), interventricular septal thickness (R^2^ = 0.23; β = 0.087, P<0.01) and posterior wall thickness (R^2^ = 0.26; β = 0.103, P<0.05), but not with end-diastolic diameter and end-systolic diameter (Table 2). The association between GDF-15 with LVH in hypertensive patients was also tested by the multivariate logistic regression model with the dependent variable as hypertension with or without LVH. After adjusted for traditional covariates age, sex, BMI, SBP, DBP, TC, LDL-C, HDL-C,TG, eGFR and glucose in the multivariate logistic regression analysis, plasma GDF-15 levels remained independently associated with LVH in hypertensive patients (p<0.01) (Table 3).

**Table 2:** Multivariable Analysis Evaluating the Association between GDF-15 and Echocardiographic variables in hypertensive patients.

variables	β	SE	R^2^	Adjusted P Value
IVSd (mm)	0.087	0.089	0.23	0.003
PWTd (mm)	0.103	0.041	0.26	0.017
EDD (mm)	0.094	0.012	0.09	0.10
ESD (mm)	0.027	0.009	0.08	0.479
LVMI (g/m^2.7^)	0.624	0.121	0.53	<0.001

Multivariate general linear was performed, adjusted by traditional covariates age, sex, BMI, SBP, DBP, TC, LDL-C, HDL-C, TG, eGFR and glucose in hypertensive patients with LVH. IVS, inter-ventricular septum; PWT, left ventricular posterior wall; EDD, end-diastolic diameter; ESD, end-systolic diameter; LVMI, left ventricular mass index. β standardized coefficients were recorded for each outcome variable. The difference is significant at P<0.05.

**Table 3:** Association of the GDF-15 with LVH in hypertensive patients.

Group	Crude OR (95% CI)	Adjusted OR (95% CI)
Hypertensive patients without LVH (n = 200)	1	1
Hypertensive patients with LVH (n = 99)	2.32(1.39–4.39) **	2.19(1.24–3.88)**

Multivariate logistic regression analysis with the dependent variable as hypertension with or without LVH was performed, adjusted by traditional covariates age, sex, BMI, SBP, DBP, TC, LDL-C, HDL-C,TG, eGFR and glucose in hypertensive patients. The difference is significant at P<0.05.*; P<0.01. **.

## Discussion

This is the first study to investigate the relations between plasma GDF-15 levels and LVH in hypertensive patients in a hospital-based sample in China. In the present study, we found that plasma GDF-15 levels were positively related to measures of LVH. Plasma levels of GDF-15 was an independent predictor of LVH even after controlling for blood pressure (SBP and DBP), as well as other conventional cardiovascular risk factors.

The process of left ventricular remodeling in hypertension is complex. The mechanisms of ventricular remodeling have not been fully clear yet, which involved in neuroendocrine factors, paracrine, and autocrine growth factors [5]. Recently, the regulation of cytokines in cardiac hypertrophy is concerning.

GDF-15 is not a cardiac-specific marker. However, GDF-15 has been recently identified as a cardioprotective cytokine in animal models. GDF-15 has also been shown to be an independent prognostic information in cardiovascular disease [23], [24]. GDF-15 are also associated to impairment of diastolic function in patients with chronic heart failure and conditions of severe disease in patients with hypertrophic cardiomyopathy (HCM), which indicated that GDF15 and may influence different processes in cardiac remodeling [25], [26]. Consistent studies have reported the association of GDF-15 with LVH in parameters of LVH in patients with myocardial infarction [27]. In the present study, we found the positive associations of plasma GDF-15 levels with several echocardiographic parameters of LVH. However, Our present findings are different from the results of a previous study demonstrating that genetic variants of GDF-15 is associated with higher GDF-15 levels and less hypertrophy in a large unselected Chinese population [28]. Several reasons might explain the different results. Firstly, endogenously determined increase of GDF-15 levels may play protective role in the process of LVH in hypertensive individuals. However, in hypertensive patients who have developed LVH, the increased levels of GDF-15 may indicate the severity of LVH. Secondly, the methodology of genetic variation association study is different from our study. Thirdly, the level of blood pressure and the extent of LVH are different in the two different populations, which might explain partially the different results.

The mechanism of GDF-15 in the pathogenesis of LV hypertrophy remains unclear. In vitro studies have shown that the GDF-15 transgenic mice with cardiac-specific overexpression have attenuated hypertrophy following the pressure overload stimulation compared to the wild type mice. Conversely, GDF-15 gene targeted mice develops cardiac hypertrophy, suggesting that GDF-15 is a cardioprotective cytokine involved in the process of cardiac hypertrophy [11]. Similarly, GDF-15 expression levels increased in infracted myocardial tissue sample of the patients died from acute myocardial infraction, indicating that endogenous GDF-15 limits myocardial tissue damage in vivo [10]. Therefore one can speculate GDF15 might be an endogenous protective mechanism that counter-regulates hypertrophy.

A recent study shows that GDF-15 as a autocrine/paracrine factor attenuates the cardiac hypertrophy in experimental models via SMAD and kinases PI3K and ERK signaling pathways, suggesting that GDF-15 provides insight into a possible mechanism involved SMAD protein activation and kinases PI3K and ERK [11]. In addition, GDF15 has recently been shown to protect ventricular remodeling against apoptotic [29]. Further experimental studies are required to address whether GDF-15 has another pressure independent effect on LV hypertrophy.

Given that small amounts of population can undermine an association study and lead to false positive results, our findings that GDF-15 levels were associated with LVH may need to be confirmed in a large sample size.

### Study Limitations

Some limitations must be considered. The patients were diagnosed as hypertension for the first time. The duration of hypertension were not known. The duration of hypertension was not adjusted in multivariable analysis, resulting in a limited statistical power. In addition, the number of patients, especially with left ventricular hypertrophy in our study was relatively small. The sample size can affect the resulting in a limited statistical power. Our findings that GDF-15 levels were associated with LVH may need to be confirmed in a large sample size.

### Conclusions

A consistent positive relation between plasma GDF-15 levels and measures of LVH in hypertensive patients were observed in this study, indicating that GDF-15 may be involved in the development of LVH in hypertension. Considering the cross-sectional design of the present study, the contribution of GDF-15 in hypertensive subjects needs to be further investigated by prospective and/or interventional studies.
